# Olink Proteomics Analysis Reveals Heterogeneous Responses to FcRn Blockade in Anti‐AChR Antibody‐Positive Myasthenia Gravis: FGF‐19 as a Novel Biomarker

**DOI:** 10.1002/acn3.70261

**Published:** 2025-11-27

**Authors:** Tiancheng Luo, Deyou Peng, Zhouao Zhang, Mingjin Yang, Xinyan Guo, Tianyu Ma, Xiaoyu Huang, Mingming Xu, Linlin Fu, Yong Zhang

**Affiliations:** ^1^ Department of Neurology The Affiliated Hospital of Xuzhou Medical University Xuzhou Jiangsu China; ^2^ First Clinical Medical College, Xuzhou Medical University Xuzhou Jiangsu China; ^3^ Central Laboratory The Affiliated Hospital of Xuzhou Medical University Xuzhou Jiangsu China; ^4^ Department of Neurology The Third People's Hospital Health Care Group of Cixi Zhejiang China; ^5^ Department of Pathogen Biology and Immunology, School of Basic Medical Sciences Xuzhou Medical University Xuzhou Jiangsu China; ^6^ Jiangsu Key Laboratory of Immunity and Metabolism Xuzhou Medical University Xuzhou Jiangsu China

**Keywords:** biomarker, efgartigimod, FGF‐19, myasthenia gravis, Olink

## Abstract

**Objective:**

This study aimed to systematically observe the clinical manifestations, immune cell subsets, and dynamic changes in serological indicators in patients with myasthenia gravis (MG) before and after efgartigimod (EFG) treatment.

**Methods:**

We analyzed the baseline data, laboratory parameters, and lymphocyte subset proportions in MG patients before and after EFG treatment. Serum levels of 92 inflammation‐related proteins were measured using Olink Target 96 proteomics in MG patients before and after EFG treatment. Validation was performed by enzyme‐linked immunosorbent assay in an expanded cohort and followed by in vitro experiments to evaluate the effects of novel biomarkers on peripheral blood mononuclear cells from MG patients.

**Results:**

Following EFG treatment, patients showed significant reductions in MG Activities of Daily Living (MG‐ADL) scores and serum IgG levels, along with remodeling of immunocyte subset distribution. Olink proteomics analysis identified six differentially expressed inflammatory cytokines, among which fibroblast growth factor‐19 (FGF‐19) exhibited the most notable downregulation. Validation in an independent cohort demonstrated that serum FGF‐19 levels were elevated and positively correlated with disease severity in MG patients. Furthermore, in EFG responders, the expression level of FGF‐19 decreased after treatment. In vitro experiments demonstrated that FGF‐19 promotes B‐cell to plasma cell differentiation in MG patients.

**Interpretation:**

EFG treatment can alleviate the clinical symptoms and decrease IgG levels in patients with MG. The downregulated expression following EFG treatment, along with the findings of in vitro experiments, indicates that FGF‐19 may not only act as a biomarker for the efficacy of EFG, but also play a role in promoting the differentiation of B cells into plasma cells, thereby offering a novel target for therapeutic interventions.

## 1 Introduction

Myasthenia gravis (MG) is an autoimmune disorder predominantly characterized by muscle weakness [1 ] . In approximately 40%–50% of patients, the disease initially manifests with ocular symptoms, called ocular MG (OMG). Among these patients, over 50% develop generalized MG (GMG) within 1–2 years [2 ] . The pathogenesis of MG is driven by autoantibodies against the neuromuscular junction. Approximately 80% of patients have antibodies against the acetylcholine receptor (AChR) [3 ] , which disrupt neuromuscular transmission through functional blockade and complement activation [4, 5].

As an antibody‐mediated disease, the pathogenesis of MG involves CD4^+^ T and B cell interactions [6]. Autoreactive CD4^+^ T cells are activated by an unknown antigen, potentially through molecular mimicry [7]. Concurrently, regulatory T cell (Treg) deficiencies facilitate the expansion of pro‐inflammatory helper T (Th)1 and Th17 cells [8, 9], which secrete cytokines, such as interferon‐γ (IFN‐γ) and interleukin (IL)‐17 which activate B cells. This leads to the differentiation of B cells into plasma cells that produce pathogenic antibodies, predominantly of the immunoglobulin G (IgG)1 and IgG3 subclasses [10].

Current standard MG management involves broad‐spectrum immunotherapy using agents such as corticosteroids, azathioprine, methotrexate, and tacrolimus [11]. Their mechanisms include suppressing antibody production, reducing circulating T cells, interfering with T/B‐cell proliferation, and inhibiting antigen‐presenting cell (APC) and T cell interactions [12]. However, these conventional therapies exhibit significant limitations including delayed onset of action, increased susceptibility to infections, broad systemic side effects, elevated malignancy risk, and drug toxicity [13].

The neonatal Fc receptor (FcRn), a major histocompatibility complex (MHC) class I‐related molecule, binds pathogenic IgG antibodies under acidic conditions, preventing their lysosomal degradation and thereby contributing to MG pathogenesis [14, 15]. Efgartigimod (EFG) is a humanized IgG1 antibody Fc fragment that acts as a natural ligand of FcRn. It exhibits higher affinity for FcRn under physiological conditions, promoting IgG catabolism and accelerating antibody clearance [16]. In the phase 2 trial of EFG, patients received efgartigimod tolerated EFG well with no adverse events, with decreasing IgG and antibody levels. Additionally, four efficacy scales (MG Activities of Daily Living [MG‐ADL], Quantitative MG scale [QMG], Myasthenia Gravis Composite disease severity [MGC], MG Quality of Life 15‐revised [MG‐QOL15r]) showed sustained improvement, indicating rapid and effective relief of MG [17]. Subsequently, the phase 3 trial also showed that EFG was generally safe and effective [18]. Therefore, EFG is a safe and effective drug for MG. However, studies on its effects on immune cells are limited, and no biomarkers indicative of its efficacy have been identified.

This study established a cohort of MG patients treated with EFG. Peripheral blood and serum samples were collected at baseline and after 1 cycle of treatment with EFG. Furthermore, we firstly used Olink proteomics detection and analysis to identify biomarkers with differential expression before and after EFG treatment. Subsequently, in vivo (enzyme‐linked immunosorbent assay [ELISA]) and in vitro experiments were conducted to validate their potential function in MG pathogenesis.

## 2 Patients and Methods

### 2.1 Patients

Initially, we recruited 28 MG patients from the Affiliated Hospital of Xuzhou Medical University to assess the efficacy of EFG, perform flow cytometry analysis, and conduct Olink analysis. Another 21 patients with MG and 13 age‐and‐sex‐matched healthy controls (HC) were recruited from our center for further validation by ELISA and for in vitro experiments. Diagnosis of MG was in accordance with the international guidelines [11]. The exclusion criteria were as follows: (1) history of infection in the last 3 months; (2) other autoimmune diseases or malignant tumors; (3) history of hyperlipidemia, coronary heart disease, cerebrovascular disease, or cognitive impairment; (4) high‐dose immunosuppressive therapy in the last 3 months, defined as prednisone > 20 mg/day; (5) mycophenolate mofetil (MMF) > 2000 mg/day; tacrolimus (TAC) > 5 mg/day; and (6) azathioprine (AZA) > 150 mg/day. The inclusion criteria for the HC were the absence of neurological, immunological, cardiovascular, autoimmune, inflammatory, or other systemic diseases. This study followed the Declaration of Helsinki and was approved by the Ethics Committee of the Affiliated Hospital of Xuzhou Medical University (XYFY2023‐KL471).

### 2.2 Treatment Schedule and Clinical Data

We systematically collected demographic and clinical data for each participant, including age, gender, Myasthenia Gravis Foundation of America (MGFA) classification, QMG score, and MG‐ADL score. EFG was administered intravenously at a dose of 10 mg/kg via a 1‐h infusion once a week per 4‐week cycle. QMG and MG‐ADL scores were recorded at baseline and at the fourth infusion. Blood samples were collected prior to initiation and after completion of the first treatment cycle for IgG quantification, immune cell subset analysis, and complete blood counts.

### 2.3 Flow Cytometry

The proportions of total B cells (defined as CD45 + CD3‐CD19 + cells), total T cells (defined as CD45 + CD3 + CD19‐cells), CD4+ T cells, CD8+ T cells, double positive T (DPT) cells (defined as CD45 + CD3 + CD4 + CD8+ cells), double negative T (DNT) cells (defined as CD45 + CD3 + CD4‐CD8‐ cells), regulatory T (Treg) cells (defined as CD45 + CD3 + CD4 + CD25 + CD127 low cells), and natural killer (NK) cells (defined as CD45 + CD3‐CD16 + CD56+ cells) were detected in 28 MG patients. The proportions of memory B cells (defined as CD45 + CD19 + CD27 + CD38‐ cells), plasmablasts (defined as CD45 + CD19 + CD27 + CD38+ cells), and plasma cells (defined as CD45 + CD19 + CD27 + CD38 + CD138+ cells) were measured in 26 MG patients. The detection of lymphocyte subsets above was conducted by flow cytometry (BD LSRF Ortessa, Franklin Lakes, NJ, USA). All data were analyzed with FlowJo V10.0.7 software (TreeStar, USA).

### 2.4 Olink Analysis

To study possible novel serum biomarkers for MG, we used the Olink Target 96 Immuno‐Oncology panel (Olink Bioscience, Uppsala, Sweden), which can detect a total of 92 human plasma proteins associated with inflammation checkpoints, chemotaxis, and tissue remodeling. The Olink assay was performed on a cohort containing 12 MG patients. The reagents use Proximity Extension Assay (PEA) technology, which allows 92 oligonucleotide‐labeled antibody probe pairs to bind to their specific target proteins if they are present in the sample. The paired oligonucleotides produce a reporter sequence after DNA polymerization, which is amplified by quantitative real‐time PCR. The data are expressed as Normalized Protein eXpression (NPX) units on a log2 scale. The raw data can be found in the Supporting Information.

### 2.5 Measurements of Serum FGF‐19

To validate the results of Olink analysis, we further collected blood samples from 21 MG patients before the first and fourth EFG administrations (during the initial phase of the treatment cycle), as well as 13 age‐ and sex‐matched HCs. Venous blood samples were obtained on an empty stomach during the early morning hours. Serum samples were subsequently extracted from the collected blood samples and centrifuged at 1000 g for 10 min, and stored at −80°C for further analysis. Serum fibroblast growth factor 19 (FGF‐19) was detected by the human FGF‐19 ELISA kit (proteintech Cat # KE00243) according to the manufacturer's instruction. We measured the optical density (OD) at 450 nm and calculated the levels of FGF‐19 using a standard curve.

### 2.6 Flow Cytometry Analysis of FGF‐19 Effects on Immune Cell Subsets

Peripheral blood mononuclear cells (PBMCs) were isolated from fresh anticoagulated blood of MG patients during active phases using Ficoll gradient centrifugation (Cat#18,061, STEMCELL Technologies). Isolated PBMCs were seeded in 24‐well plates and treated with 100 ng/mL recombinant FGF‐19 protein (Cat no: HZ‐1330, Proteintech). After 5 days of culture for B cells and 3 days for T cells, differentiation was assessed. Cultured cells were stained with a mixture of anti‐CD19‐APC (Cat#302,212), anti‐CD27‐BV421 (Cat#356,418), anti‐IgD‐PE (Cat#348,204), anti‐CD38‐FITC (Cat#303,504), and anti‐CD138‐APC‐Cy7 (Cat#356,528) (all from Biolegend). For intracellular cytokine staining, cultured PBMCs were stimulated with 2 μL eBioscience Cell Stimulation Cocktail (Cat#00–4970‐03, Invitrogen) for 5 h. Cell surface markers were stained with anti‐CD3‐FITC (Cat#317,306, Biolegend) and anti‐CD4‐BV421 (Cat#562,424, BD Biosciences), followed by fixation and permeabilization using eBioscience Intracellular Fixation and Permeabilization Buffer Kit (Cat#88–8824‐00, Invitrogen), then stained with anti‐IFN‐γ‐Percp‐Cy5.5 (Cat#506,528), anti‐IL‐4‐PE‐Cy7 (Cat#500,824), and anti‐IL‐17A‐PE (Cat#512,306) (all from Biolegend). For Treg intracellular staining, cell surface markers were stained with anti‐CD4‐FITC (Cat#317,408) and anti‐CD25‐APC (Cat#385,606) (Biolegend), followed by fixation and permeabilization using BD Cytofix/Cytoperm TM Reagent (Cat#562,574, BD Biosciences), then stained with anti‐FOXP3‐PE (Cat#320,208, Biolegend). All samples were analyzed by flow cytometry as described.

### 2.7 Statistical Analysis

Statistical Package for the Social Sciences (SPSS 26.0) and GraphPad Prism software 10.0 were used. Demographic, clinical, and laboratory characteristics of study subjects are presented as mean ± standard deviation or proportions as appropriate. Comparison between two groups used paired sample t‐tests (normal distribution), Wilcoxon paired test (non‐normal distribution), and independent sample t‐tests. Spearman correlation analysis was performed to study relationships between variables. Receiver operating characteristic (ROC) curve analysis was performed to evaluate the predictive value of FGF‐19 for MG. Statistical significance was considered when *p* < 0.05. No adjustment for multiple comparisons was made due to the exploratory nature of this study.

## 3 Results

### 3.1 Clinical Response in Patients Receiving Efgartigimod Treatment

**FIGURE 1:**
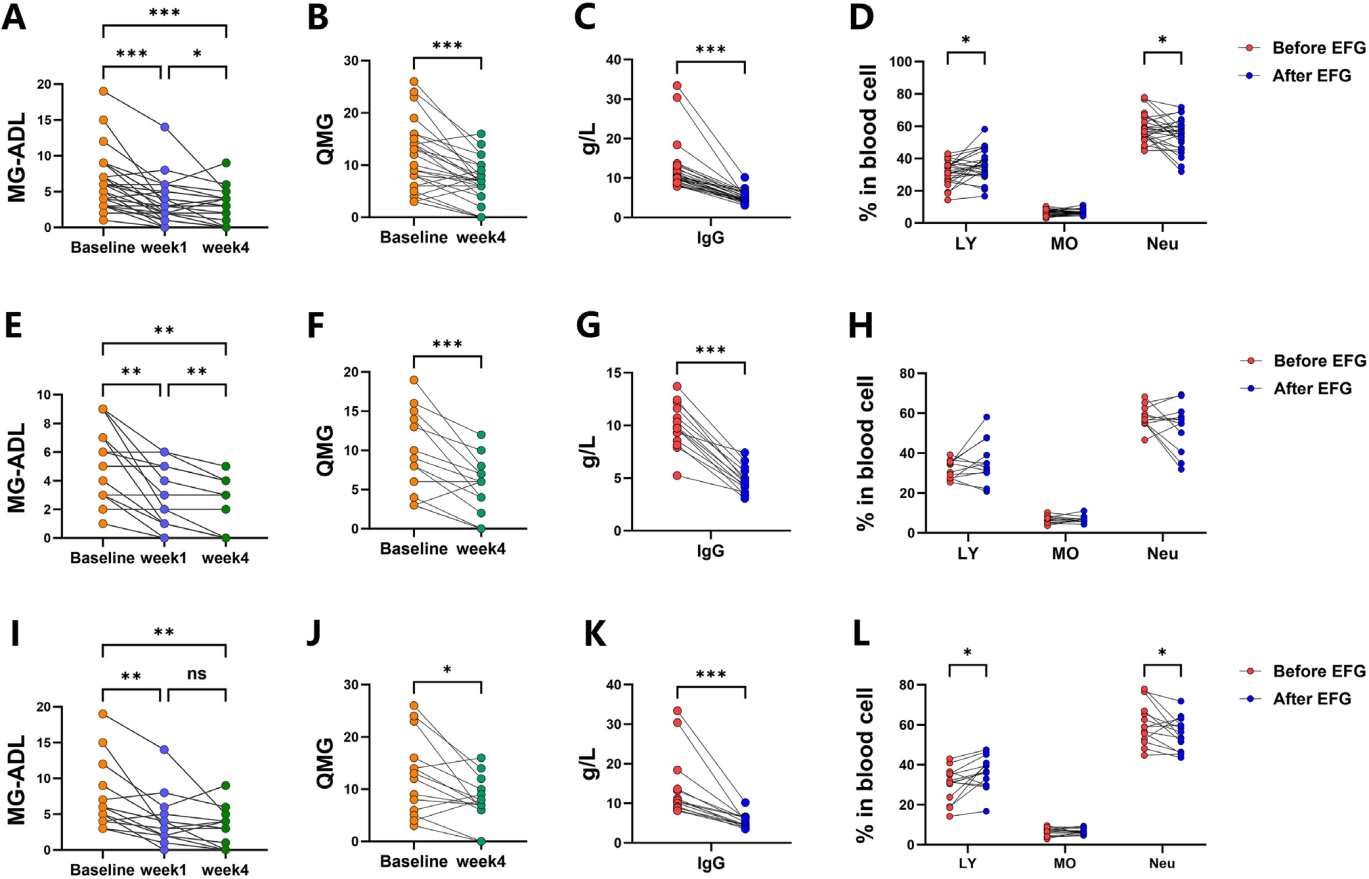
Clinical and hematological responses to EFG treatment in MG patients. (A) MG‐ADL scores across treatment phases (baseline, week 1, cycle 1 end; *n* = 28). (B–D) QMG scores, serum IgG, and blood cell frequencies (LY, MO, Neu) at baseline versus cycle 1 end (*n* = 28). (E–H) Changes under EFG monotherapy (*n* = 14). (I–L) Changes with EFG plus corticosteroids (*n* = 14). EFG, efgartigimod; IgG, immunoglobulin G; LY, lymphocytes; MO, monocytes; Neu, neutrophils.

We evaluated the MG‐ADL scores at baseline, 1 week, 4 weeks after the initial EFG infusion, QMG scores at baseline and at the end of the first cycle (week 4), as well as serum IgG levels and total blood cell counts (including lymphocytes, monocytes, and neutrophils) at baseline and at the end of the first cycle in 28 patients with MG (cohort 1). The demographic data of those patients were listed in Table 1. The mean baseline MG‐ADL score of the 28 MG patients was 6.2, which decreased to 3.4 one week after the initial EFG infusion and to 2.4 at the end of the first cycle (Figure 1A). The QMG score decreased from 11.0 at baseline to 6.5 at week 4 (Figure 1B). Levels of serum IgG decreased during the first cycle which reduced to approximately 42% of baseline levels at week 4 (Figure 1C). Compared to baseline, the percentage of lymphocytes increased and the frequency of neutrophils decreased at the end of the first cycle, while monocytes showed no statistically significant changes (Figure 1D). Among 28 patients, 14 patients received EFG monotherapy (who had poor therapeutic effects on pyridostigmine and were thus treated with efgartigimod without any other immunosuppressant); their mean MG‐ADL score decreased from 5.3 to 2.8 at week 1 and 2.0 at week 4 (Figure 1E). The mean QMG score decreased from 9.4 to 5.1 (Figure 1F). The IgG levels decreased to approximately 49% of pre‐treatment levels (Figure 1G). No significant changes were observed in lymphocytes, neutrophils, nor monocytes (Figure 1H). Fourteen patients received combination therapy (14 received prednisone and 4 received prednisone combined with tacrolimus). Their mean MG‐ADL score decreased from 7.4 to 4.1 at week 1 and 3.0 at week 4, and the mean QMG score decreased from 12.6 to 7.8 (Figure 1I,J). The IgG levels were 38% of pre‐treatment levels (Figure 1K). Unlike the monotherapy group, patients receiving EFG combined with immunosuppressive therapy showed significant changes in lymphocytes and neutrophils (Figure 1L).

**TABLE 1:** Characteristics of myasthenia gravis patients and healthy controls.

Characteristic	Cohort 1	Cohort 2		
	AChR‐MG (*n* = 28)	AChR‐MG (*n* = 21)	HC (*n* = 13)	*p*
Age, years (±SD)	58.2 ± 14.2	51.2 ± 7.9	54.6 ± 11.1	0.200
Sex, *n*				0.290
Male	11	9	8	
Female	17	12	5	
Duration, years (±SD)	1.7 ± 1.6	2.5 ± 3.1		
MG subgroups				
EOMG	7	6		
LOMG	21	15		
Thymoma	5	6		
MGFA classification				
I	4	1		
II	9	7		
III	13	9		
IV	2	4		
Baseline MG‐ADL score (±SD)	5.9 ± 4.5	6.5 ± 4.1		
Ongoing therapies				
EFG alone	14	21		
EFG with prednisone	14	0		

Abbreviations: AChR, acetylcholine receptor; EFG, efgartigimod; ELISA, enzyme‐linked immunosorbent assay; EOMG, early‐onset myasthenia gravis; GMG, generalized myasthenia gravis; HC, healthy control; LOMG, late‐onset myasthenia gravis; MG‐ADL, Myasthenia Gravis Activities of Daily Living; MGFA, Myasthenia Gravis Foundation of America; OMG, ocular myasthenia gravis; SD, standard deviation.

### 3.2 Changes in Lymphocyte Subsets Following Efgartigimod Treatment

**FIGURE 2:**
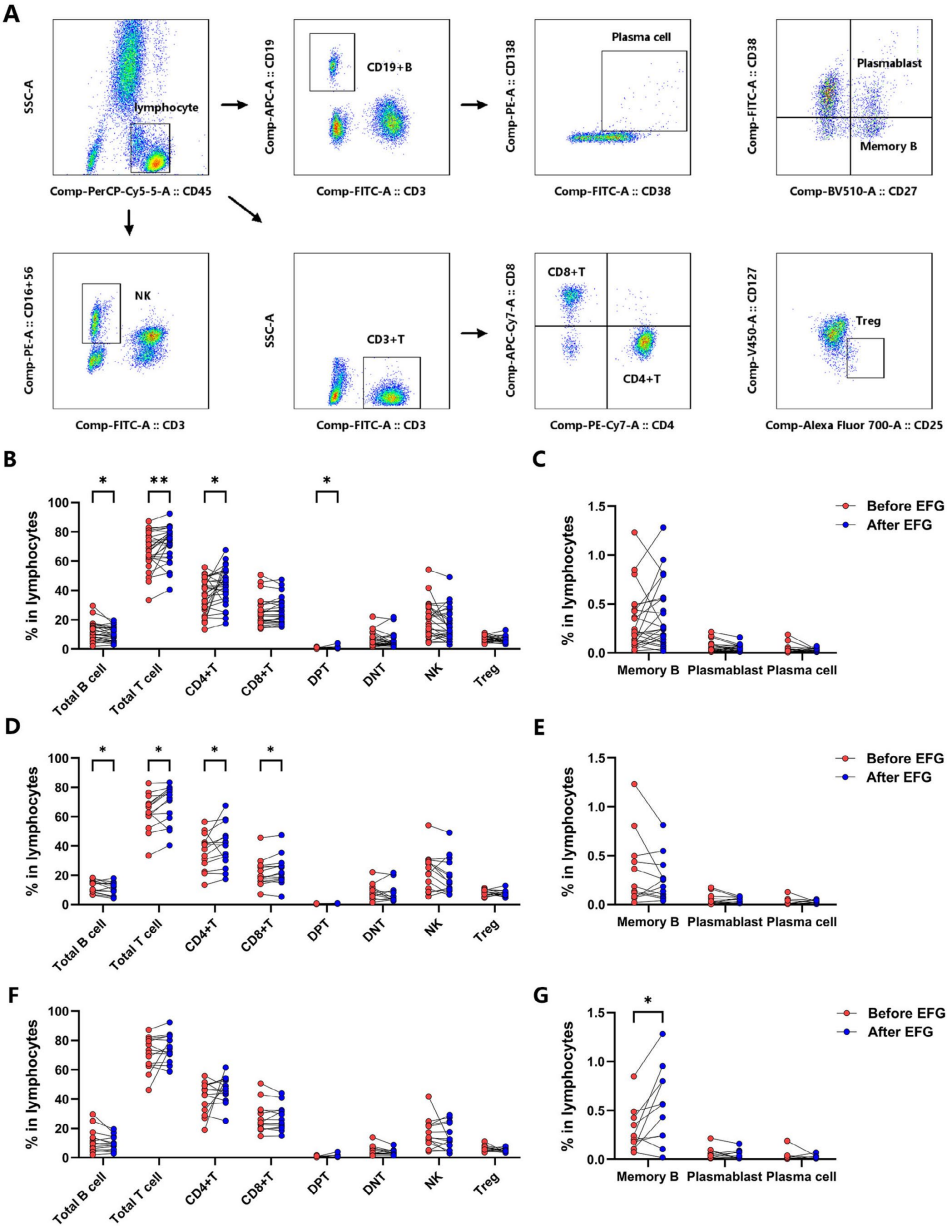
Lymphocyte subset alterations following EFG treatment. (A) PBMC gating strategy. (B, C) Subset frequency changes post‐treatment (*n* = 28). (D, E) Monotherapy subset dynamics (*n* = 14). (F, G) Combination therapy subset dynamics (*n* = 14). DNT, double‐negative T cells; DPT, double‐positive T cells; EFG, efgartigimod; PBMC, peripheral blood mononuclear cell.

We assessed the effects of efgartigimod treatment on circulating immune cells in MG patients using flow cytometry (Figure 2A). After one treatment cycle, the frequency of CD19+ B cells decreased (*p* = 0.015, Figure 2B), while the frequency of CD3+ T cells increased (*p* = 0.002, Figure 2C). When evaluating T‐cell subsets, the frequencies of CD4+ T cells (*p* = 0.015) and double‐positive T (DPT) cells (*p* = 0.029) increased following efgartigimod treatment. However, no significant changes were observed in the frequencies of CD8+ T cells, DNT cells, NK cells, Treg cells, memory B cells, plasmablasts, or plasma cells (Figure 2B,C). Changes in lymphocyte subsets were generally consistent in patients receiving EFG monotherapy (Figure 2D,E). Notably, the frequency of CD8+ T cells was significantly upregulated (*p* = 0.025), while the change in DPT cells was not significant in patients receiving EFG monotherapy (Figure 2D,E). Compared to patients treated with efgartigimod alone, combination therapy with prednisone seemed to lead to an increased frequency of memory B cells (*p* = 0.017). However, no statistically significant differences were observed in the changes of other lymphocyte subsets compared to the monotherapy group (Figure 2F,G).

### 3.3 Serological Changes and DEP Enrichment Analysis in MG Patients Treated With Efgartigimod

**FIGURE 3:**
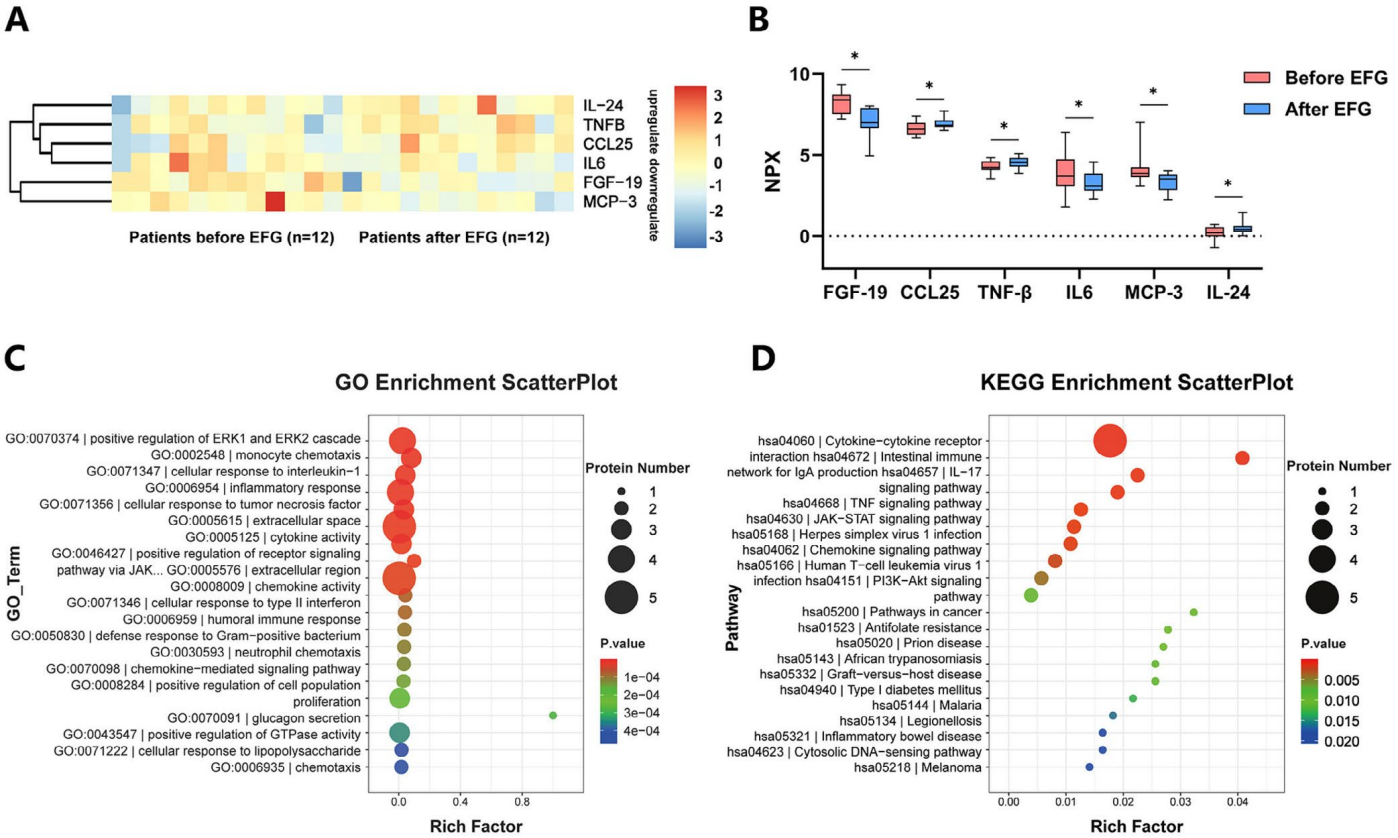
Proteomic profiling and pathway enrichment pre/post EFG treatment. (A) Heatmap of 6 inflammation‐related proteins. (B) Differentially expressed proteins. (C, D) Enriched GO/KEGG pathways. GO, Gene Ontology; KEGG, Kyoto Encyclopedia of Genes and Genomes; NPX, normalized protein expression.

To investigate the potential immunomodulatory effects of efgartigimod in MG patients, we are using the Olink assay to detect inflammation‐related proteins. Serum samples from 12 patients treated with efgartigimod monotherapy were analyzed before and after treatment. The results showed that, compared to baseline, the serum levels of FGF‐19 (*p* = 0.0038), IL‐6 (*p* = 0.0137), and monocyte chemoattractant protein (MCP)‐3 (*p* = 0.0200) were downregulated after EFG treatment, while C‐C motif chemokine ligand (CCL)‐25 (*p* = 0.0079), TNF‐β (*p* = 0.0109), and IL‐24 (*p* = 0.0431) were upregulated (Figure 3A,B). Differentially expressed proteins (DEPs) identified from the Olink analysis were subjected to enrichment analysis. Enriched proteins were associated with several GO terms, including extracellular space, extracellular region, and inflammatory response. Enriched KEGG pathways were primarily concentrated in cytokine‐cytokine receptor interaction (Figure 3C,D).

### 3.4 Serum FGF‐19 Elevation in MG With Clinical Severity Correlation and Reduction Following EFG Treatment

**FIGURE 4:**
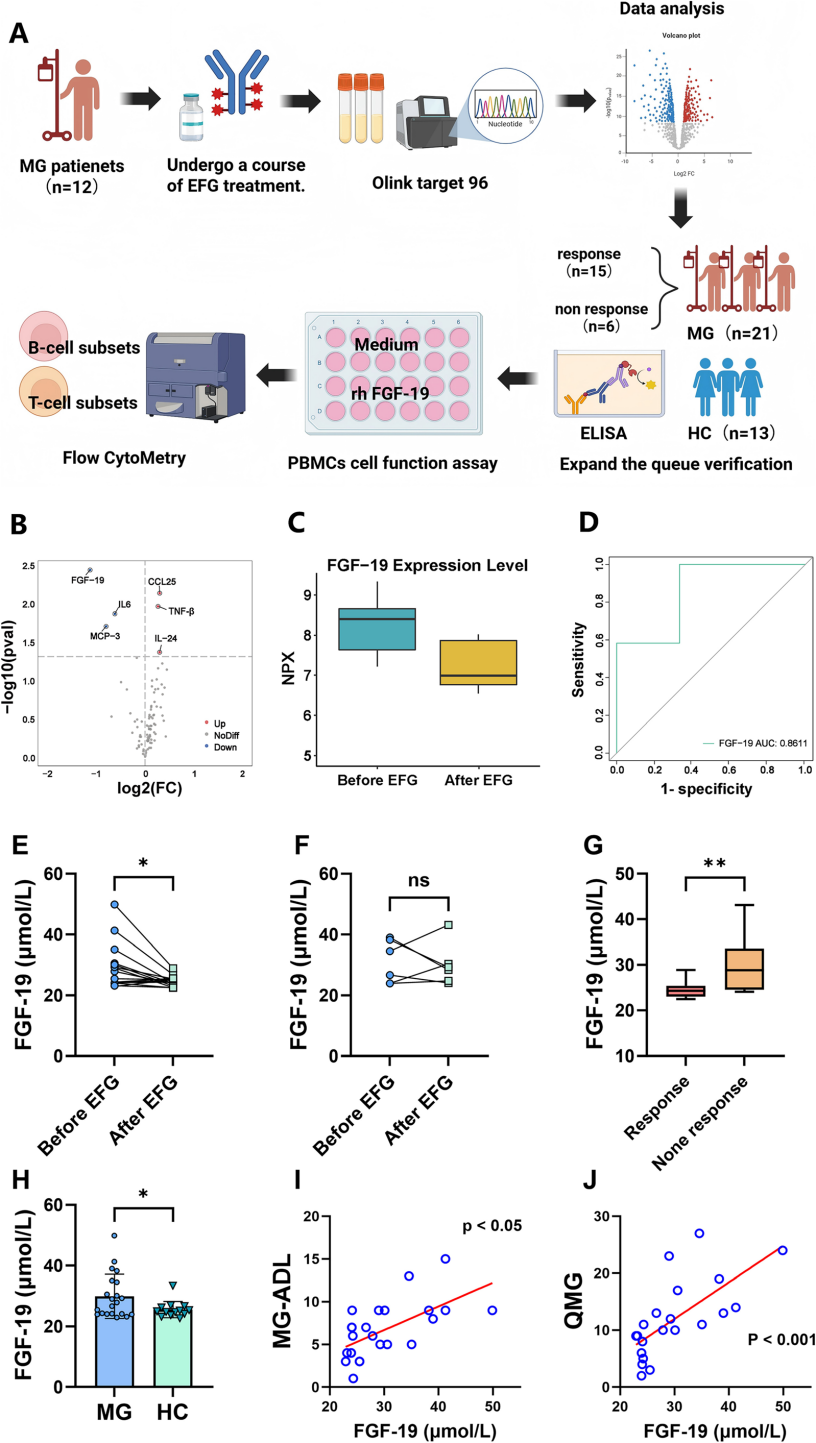
FGF‐19 as a biomarker in EFG‐treated MG. (A) Flowchart of the study (B) Volcano plot of FGF‐19 changes. (C) Pre/post‐treatment levels. (D) Diagnostic ROC curve. (E, F) Responder (*n* = 15) versus non‐responder (*n* = 6) dynamics. (G) Post treatment comparison. (H) MG (*n* = 21) versus healthy controls (*n* = 13). (I, J) Clinical score correlations. *Responders: ≥ 2‐point MG‐ADL improvement sustained ≥ 4 weeks.

To identify biomarkers for evaluating the efficacy of EFG treatment in MG patients, we selected FGF‐19, which exhibited the most significant changes post‐treatment, for further analysis (Figure 4A,B). Olink analysis showed serum FGF‐19 NPX decreased from 8.23 to 7.11 after EFG treatment (*p* = 0.0038). ROC curve analysis was used to evaluate the ability of FGF‐19 to discriminate between pre‐treatment and post‐treatment status, with an area under the curve (AUC) of approximately 0.86 (Figure 4C,D). We established a new cohort including 21 MG patients and 13 HCs (Table 1). Among them, 15 were MG‐ADL responders and 6 were non‐responders after EFG treatment (MG‐ADL responder was defined as a ≥ 2‐point reduction in MG‐ADL score sustained for ≥ 4 consecutive weeks) [18]. In the responder group, FGF‐19 levels decreased significantly after EFG (*p* < 0.05, Figure 4E), while there was no significant change in the non‐responders group (*p* > 0.05, Figure 4F). After EFG, the FGF‐19 levels were higher in non‐responders than in responders (*p* < 0.05, Figure 4G). Compared with healthy controls, serum FGF‐19 levels were higher in MG patients before treatment (*p* < 0.05, Figure 4H). Correlation analysis demonstrated positive correlations between pre‐treatment serum FGF‐19 levels and MG‐ADL scores (*r* = 0.62, *p* < 0.05) as well as QMG scores (*r* = 0.68, *p* < 0.001) (Figure 4I,J).

### 3.5 FGF‐19 Promotes Plasma Cell Differentiation in PBMCs of MG Patients

**FIGURE 5:**
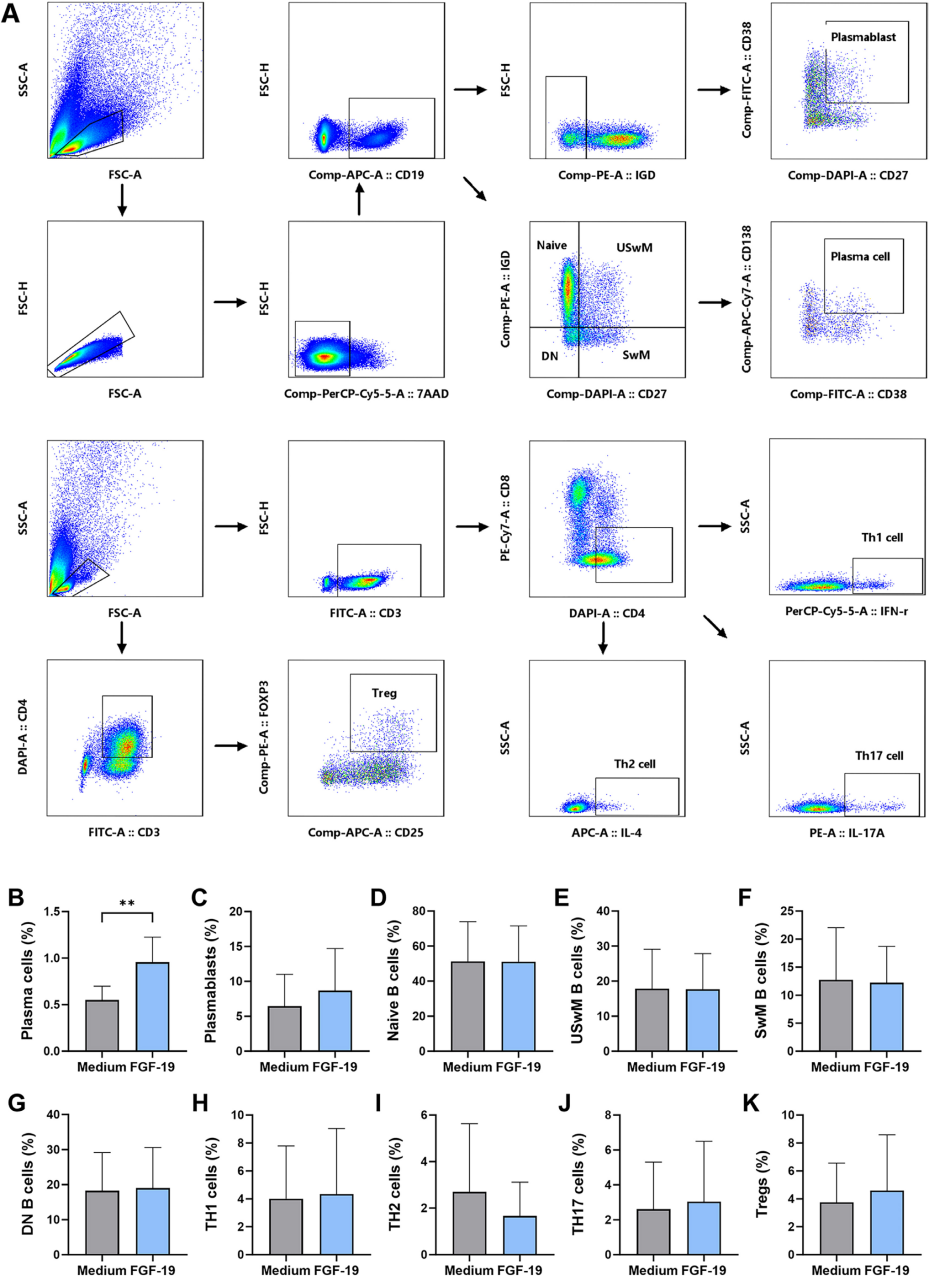
FGF‐19 promotes plasma cell differentiation. (A) PBMC gating strategy. (B–K) Subset frequencies with/without FGF‐19 stimulation (*n* = 7). DNB, double‐negative B cells; SwMB, switched memory B cells; USwMB, unswitched memory B cells.

To further determine the effect of FGF‐19 on immune cells in MG patients, PBMCs from 7 newly diagnosed MG patients were cultured with recombinant FGF‐19 protein in our experimental system. Immune cell subsets were analyzed by flow cytometry (Figure 5A). Results showed that FGF‐19 promoted plasma cell formation (*p* < 0.005, Figure 5B), while no significant effects were observed on CD4+ T cell subsets (Th1, Th2, Th17, Treg) or other B cell subsets (naïve B cells, switched memory B cells (SwMB), unswitched memory B cells (USwMB), double‐negative B cells (DNB), and plasmablasts (Figure 5C–K)).

## 4 Discussion

MG is a B cell‐mediated autoimmune disorder where pathogenic autoantibodies are primarily produced by plasma cells targeting specific self‐antigens [19]. Concurrently, B cells exacerbate disease progression through their antigen‐presenting function [20], activating T cells to secrete pro‐inflammatory cytokines [21]. While EFG, a safe and effective FcRn antagonist, has demonstrated efficacy in reducing pathogenic antibodies in MG [18], its impact on immune cells particularly B cells remains incompletely understood. Our study revealed that CD19^+^ B cells were downregulated following the first treatment cycle of EFG, correlating with clinical improvement. To further investigate EFG's immunomodulatory effects, we employed Olink proteomics to identify novel biomarkers that may inform disease diagnosis and therapeutic response.

FcRn and classical Fcγ receptors (FcγRs) are highly expressed on immune cells [22]. Their synergistic interaction with IgG forms soluble or cell‐associated immune complexes, stimulating antigen‐presenting cells to produce inflammatory mediators [23]. Moreover, these complexes are processed via class I HLA molecules to activate CD8^+^ cytotoxic T cells or via class II HLA molecules to activate CD4^+^ helper T cells. This cascade amplifies B cell responses, driving plasma cell differentiation and pathogenic IgG secretion, thereby perpetuating MG progression [24]. Our data suggest that EFG's blockade of FcRn reduces circulating IgG and thereby modulates B cell dynamics. This effect was more pronounced in EFG monotherapy than in combination with corticosteroids, corroborating prior evidence that corticosteroids upregulate CD19^+^ B cells [25]. Therefore, how to combine steroid hormones with EFG to maximize therapeutic efficacy would be a new challenge in MG treatment.

To delineate serological changes under EFG therapy, we performed Olink proteomic analysis of MG patient sera. Proteomic analysis of MG patient serum revealed significant serological changes following EFG treatment, accompanied by disease remission, characterized by a marked reduction in FGF‐19, IL‐6, and MCP‐3, and an increase in TNF‐β, CCL‐25, and IL‐24. The downregulated factors, IL‐6 and MCP‐3, are known to drive inflammation by promoting Th17 cell differentiation and recruiting monocytes and T cells to inflammatory sites, respectively [26, 27]. Particularly, IL‐6 has been reported to promote B cell differentiation and contribute to the pathogenesis of MG [28]. Upregulated IL‐24 may attenuate inflammation and immune responses by suppressing pathogenic Th17 cells and inhibiting B cell differentiation into plasma cells [29, 30]. These dynamic changes suggest that EFG likely exerts its therapeutic effects by modulating inflammatory mediators. Notably, FGF‐19 exhibited the most significant reduction. FGF‐19 is a member of the endocrine FGF subfamily, produced in the gut and acting via FGFR receptors [31], and displays context‐dependent roles in inflammation: it may exert anti‐inflammatory effects yet promote hepatic inflammation in alcoholic liver disease and correlate with poor prognosis in hepatocellular carcinoma [32, 33, 34, 35]. Given the limited evidence on its role in autoimmunity, we measured serum FGF‐19 levels in MG patients and compared them with healthy controls from the same period. We found that serum FGF‐19 levels were elevated in MG patients compared to healthy individuals and decreased after EFG treatment. Crucially, FGF‐19 reduction was significant only in MG‐ADL responders treated with EFG, suggesting its utility as a predictive biomarker for EFG efficacy.

So far, the role of FGF‐19 in autoimmunity remains undefined. Previous studies have shown that FGF‐19 binding to its receptor complex (FGFR/β‐Klotho) can effectively induce the phosphorylation of extracellular signal‐regulated kinase 1/2 (ERK1/2) [36], whose activation is a driver of terminal B cell differentiation into plasma cells [37], which serve as the main source of pathogenic autoantibodies in MG [38]. Specifically, our study also found that FGF‐19 promotes the differentiation of B cells into plasma cells in MG patients. These mechanisms suggest that FGF‐19 may be involved in the pathogenesis of MG.

This study has several limitations. The observation period for patients was relatively short, and there was a lack of a placebo control group. Furthermore, we exclusively enrolled patients who were seropositive for anti‐AChR antibodies, meaning those with MuSK‐positive or double‐seronegative myasthenia gravis (MG) were not included for experimental validation. Additionally, the downstream mechanisms by which EFG influences lymphocyte subsets and serum FGF‐19 levels were not thoroughly investigated. Longer‐term and more in‐depth studies are currently underway, and we will further analyze these results.

## 5 Conclusion

In summary, our findings demonstrate that EFG treatment reduced total B‐cell counts while increasing total T‐cell and CD4+ T‐cell counts, with these changes being influenced by a combination of steroid hormones. Serum analysis of patients revealed elevated FGF‐19 expression in MG patients, which decreased following EFG treatment and correlated with EFG efficacy, suggesting its potential as a biomarker for assessing EFG treatment response in myasthenia gravis. Moreover, FGF‐19 promoted B‐cell differentiation into plasma cells, providing novel insights into the pathogenesis and treatment of myasthenia gravis.

## Author Contributions

All authors have made a substantial contribution to the design, data collection, and analysis of the research and the drafting of the manuscript and reviewed and accepted the contents of the manuscript prior to its submission. Tiancheng Luo, Deyou Peng, Xiaoyu Huang, and Zhouao Zhang: conception and design of the study, acquisition, and analysis of data and drafting or revising a significant portion of the manuscript or figures. Mingjin Yang, Xinyan Guo, and Tianyu Ma: acquisition and analysis of data. Yong Zhang, Xiaoyu Huang, Linlin Fu, and Mingming Xu: drafting or revising a significant portion of the manuscript or figures.

## Funding

The author(s) declare that financial support was received for the research and/or publication of this article. This work was supported by the Medical Research Project of Jiangsu Provincial Health Commission (M2022118), the Natural Science Foundation of Jiangsu Province (BK20231158), the Youth Medical Science and Technology Innovation Project of Xuzhou Municipal Health Commission (XWKYHT20240110), the Hospital‐level Scientific Research Project of the Affiliated Hospital of Xuzhou Medical University (2024ZL30), the Construction Project of High Level Hospital of Jiangsu Province (GSPJS202503) and Cixi Public Welfare Special Project (CN2024026).

## Conflicts of Interest

The authors declare no conflicts of interest.

## Supporting information


**Data S1:** Supporting Information

## Data Availability

The data that support the findings of this study are available on request from the corresponding author. The data are not publicly available due to privacy or ethical restrictions.
